# Comprehensive analysis of intervention and control studies for the computational identification of dengue biomarker genes

**DOI:** 10.1371/journal.pntd.0012914

**Published:** 2025-03-18

**Authors:** Jibon Kumar Paul, Mahir Azmal, Tasnim Alam, Omar Faruk Talukder, Ajit Ghosh

**Affiliations:** Department of Biochemistry and Molecular Biology, Shahjalal University of Science and Technology, Sylhet, Bangladesh; Aarupadai Veedu Medical College & Hospital, INDIA

## Abstract

Dengue fever, caused by the dengue virus (DENV), presents a significant global health concern, with millions of cases reported annually. Despite significant progress in understanding Dengue fever, effective prognosis and treatment remain elusive due to the complex clinical presentations and limitations in current diagnostic methods. The virus, transmitted primarily by the *Aedes aegypti* mosquito, exists in four closely related forms, each capable of causing flu-like symptoms ranging from mild febrile illness to severe manifestations such as plasma leakage and hemorrhagic fever. Although advancements in diagnostic techniques have been made, early detection of severe dengue remains difficult due to the complexity of its clinical presentations. This study conducted a comprehensive analysis of differential gene expression in dengue fever patients using multiple microarray datasets from the NCBI GEO database. Through bioinformatics approaches, 163 potential biomarker genes were identified, with some overlapping previously reported biomarkers and others representing novel candidates. Notably, AURKA, BUB1, BUB1B, BUB3, CCNA2, CCNB2, CDC6, CDK1, CENPE, EXO1, NEK2, ZWINT, and STAT1 were among the most significant biomarkers. These genes are involved in critical cellular processes, such as cell cycle regulation and mitotic checkpoint control, which are essential for immune cell function and response. Functional enrichment analysis revealed that the dysregulated genes were predominantly associated with immune response to the virus, cell division, and RNA processing. Key regulatory genes such as AURKA, BUB1, BUB3, and CDK1 are found to be involved in cell cycle regulation and have roles in immune-related pathways, underscoring their importance in the host immune response to Dengue virus infection. This study provides novel insights into the molecular mechanisms underlying Dengue fever pathogenesis, highlighting key regulatory genes such as AURKA and CDK1 that could serve as potential biomarkers for early diagnosis and targets for therapeutic intervention, paving the way for improved management of the disease.

## Author Summary

Dengue fever, caused by the dengue virus (DENV), remains a significant global health issue with millions of cases annually. Accurate diagnosis and early identification of severe cases are critical challenges. This study aimed to identify potential biomarker genes associated with dengue fever by analyzing gene expression data from multiple studies. Using advanced bioinformatics tools, 163 potential biomarker genes were identified. Notably, genes such as AURKA, BUB1, and CDK1, which are involved in cell cycle regulation and immune responses, were significantly dysregulated. Functional enrichment analysis revealed that these genes are primarily associated with immune response, cell division, and RNA processing pathways, such as mitotic nuclear division and chromosome segregation. The identification of these key regulatory genes not only advances our understanding of the molecular mechanisms underlying dengue fever but also provides promising targets for developing early diagnostic methods and therapeutic interventions. These insights could lead to more effective management of dengue, potentially reducing its severe manifestations and associated mortality.

## Introduction

Dengue virus (DENV) is a type of flavivirus that is mainly transmitted by the female *Aedes aegypti* mosquito, predominantly found in urban areas. The dengue virus has been recognized for centuries, with early dengue-like symptoms documented in the 17th century [1] and confirmed cases appearing by the late 18th century [2]. After a non-infected mosquito bites an infected human, the incubation period within the mosquito lasts for 4-10 days [3]. Following the incubation period, the vector could transmit the virus throughout its entire lifespan. At present, this viral fever is a substantial global health concern, with an approximate yearly incidence of 390 million illnesses across the globe. The condition is widespread in more than 100 nations, especially in tropical and subtropical areas, where almost 50% of the global population is vulnerable [4]. Dengue virus (DENV) has four distinct serotypes: DENV-1, DENV-2, DENV-3, and DENV-4. Each serotype can cause a spectrum of clinical outcomes, ranging from mild febrile illness to severe conditions such as dengue hemorrhagic fever (DHF) and dengue shock syndrome (DSS). Secondary infection with a different serotype significantly increases the risk of severe disease due to a phenomenon known as antibody-dependent enhancement (ADE), where pre-existing antibodies from the first infection facilitate viral entry into cells during subsequent infections. Understanding the clinical implications of each serotype is crucial for effective disease management and the development of targeted therapeutic interventions. All four DENV serotypes evolve in subtropical Asia, Africa, Europe, and North and South America. All ages can get a flu-like illness from DENV, but more severe symptoms like plasma leakage are also common [5,6]. A fever of 104°F, aches behind the eyes, severe headache, muscle/joint pain, vomiting, inflamed glands, and rash are typical infection symptoms [7]. These symptoms appear 4–10 days after a mosquito bite and last up to 7 days [8]. Plasma leaking, fluid accumulation, ascites, pleural effusions, severe bleeding, low platelets, and organ impairment can make severe dengue fever (DF) life-threatening [9]. While DENV-2 and DENV-4 are often associated with more severe manifestations such as DHF, all four serotypes have the potential to cause both mild and severe disease forms, depending on various host and viral factors. These serotypes are predominantly transmitted by Aedes mosquitoes [10].

Dengue disease has a range of clinical manifestations, encompassing mild febrile sickness as well as severe and potentially life-threatening forms like DHF and DSS [11]. DHF and DSS are distinguished by the presence of plasma leakage, thrombocytopenia, and hemorrhagic conditions, resulting in substantial illness and death [9]. Diagnosing dengue fever is difficult since its clinical symptoms are not distinct, and it resembles other febrile disorders, including malaria and chikungunya [12]. Accurate diagnosis necessitates laboratory confirmation by molecular or serological assays [13]. Timely identification and expeditious clinical intervention are crucial in mitigating the likelihood of severe complications and mortality linked to dengue fever [14].

The utilization of biomarkers as preemptive indicators in the assessment of illness susceptibility has gained significant traction in recent years, particularly in infectious diseases. Biomarkers are measurable characteristics that indicate common biological processes, disease-causing processes, or the body’s response to treatment with drugs [15]. Specifically, in infectious diseases, biomarkers can aid in early diagnosis, predict disease severity, and guide therapeutic decisions [16]. A systematic review and meta-analysis identified key biomarkers predictive of severe dengue manifestations, including elevated levels of CRP, AST, IL-8, and decreased albumin levels for DHF, and increased levels of VCAM-1, syndecan-1, AST, and CRP for severe dengue (SD). These findings highlight the role of early acute inflammation and hepatic involvement in the progression of dengue severity and suggest the potential for further validation of numerous additional biomarkers associated with DHF and SD [17]. The utilization and recognition of biomarkers in the medical and clinical domains have a significant influence on society. A recent study delves into the historical context, varied definitions, classifications, distinctive features, and the methodology for identifying biomarkers [18]. Additionally, the possible use of biomarkers in diagnosing, predicting the course, and treating different diseases during the past decade. The purpose of the study is to encourage readers to investigate new possibilities in the field of biomarker research and development [19]. Current scientific investigations have been primarily dedicated to comprehending the etiology of dengue fever by employing diverse molecular methodologies [20], such as the examination of hub gene prediction, cluster formation, protein-protein interaction (PPI) networks, gene co-expression networks, and molecular pathways [21]. These methodologies offer significant perspectives on the intricate interaction between viral and host elements that contribute to the advancement of diseases [22].

Although PPI and gene co-expression networks are characterized by their static nature, they do not account for dynamic changes over time or in response to varying conditions, but they still provide valuable insights into the steady-state molecular interactions during dengue virus infection [23]. Research has emphasized the usefulness of these studies in comprehending the interactions between viruses and hosts, regulating the immune response of the host, and identifying possible targets for therapeutic interventions [24–27].

The research aims to identify biomarker genes associated with dengue fever by integrating differential gene expression data and constructing functional networks. Significant differentially expressed genes (DEGs) were identified and analyzed to reveal key molecular pathways involved in immune response, viral replication, and cellular stress during dengue infection. The findings highlight potential biomarkers for improved diagnostics and targeted therapies. These insights into the molecular mechanisms of dengue fever can aid in the development of precise diagnostic tools, identification of novel therapeutic targets, and stratification of patients based on risk, ultimately improving clinical decision-making and reducing severe outcomes like DHF and DSS.

## Materials and methods

### Study design

**Fig 1:**
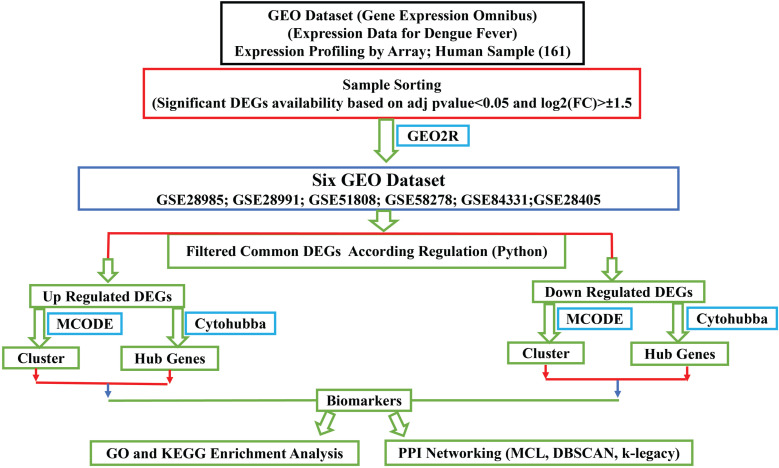
A Schematic illustration of the overall workflow. Initially, significant DEGs are filtered based on p-value and log2 (FC) criteria. Six GEO datasets (GSE28985, GSE28991, GSE51808, GSE58278, GSE84331, GSE28405) are analyzed, and common DEGs are identified. Up-regulated and down-regulated DEGs are processed separately using MCODE for clustering and cytoHubba for hub genes. The identified biomarkers undergo GO and KEGG enrichment analysis and PPI networking to determine their biological significance and interactions.

The study design was structured to identify potential biomarker genes associated with dengue fever through a comprehensive bioinformatics analysis of multiple microarray datasets obtained from the NCBI GEO database. Six GEO datasets were selected based on their relevance to dengue infection and the availability of raw expression data for differential expression analysis. The datasets spanned different cell types and sample sizes, ensuring a broad representation of gene expression patterns. The specific bioinformatics tools employed included GEO2R for differential expression analysis, Cytoscape’s (v3.10.0) cytoHubba, and MCODE (v2.0.0) plugins were used to identify biomarker genes. cytoHubba is a versatile tool that ranks nodes (genes) in a network based on 12 various algorithms, such as Degree, MCC, and Closeness bottleneck, EPC, DMNC, Stress, Betweenness, Radiality, Cluster Coefficient etc., making it ideal for identifying hub genes that are central to the network’s function. MCODE, on the other hand, detects densely connected regions within the network, which are often indicative of biologically relevant gene clusters. In a PPI network, MCODE scores nodes according to their local density before identifying clusters. Then, it chooses nodes with high scores as seeds to start clusters. It comprises nodes that have a sufficient density using the Node Score Cutoff. Clusters are refined by Haircut by eliminating singly connected nodes and expanded by Fluff by adding nodes connected to many cluster members. Cluster size is limited by Max Depth from Seed, which restricts node inclusion distance, and by the K-Core parameter, which guarantees a minimum connectivity level. The Weishengxin bioinformatics platform (https://bioinformatics.com.cn/) for pathway and GO enrichment analysis. These tools were chosen for their robust algorithms and established utility in gene expression and network analysis, ensuring the reliability of the findings (Fig 1).

### Microarray data

The Gene Expression Omnibus is a publicly accessible and operational genomics database that houses large-scale gene expression data, including chips and microarrays. In this study, microarray data were downloaded with a search of **“dengue”** with a parameter of “**Expression profiling by array**” for analysis with ***“Homo sapiens”*** in the data filtration (Table 1). Each data set contains samples from healthy control and infected individuals. The different cells were studied for the comprehensive study of differential gene expression and their co-relation.

**Table 1:** Summary of gene expression profiling studies containing gene expression profiles for six datasets.

Sl no	Study	Sample	Control	Case	Organ/Tissue	Reference
1	GSE28985	96	12	84	U937 cell line	https://www.ncbi.nlm.nih.gov/geo/query/acc.cgi?acc=GSE28985
2	GSE28991	33	11	22	Blood	[28]
3	GSE51808	56	9	47	Whole Blood	[29]
4	GSE58278	18	6	12	Monocyte-derived denritic cells	[30]
5	GSE84331	12	5	7	CD8 T cells	[31]
6	GSE28405	119	26	93	Whole Blood	[32]

### Data processing

GEO2R (Version: R 4.2.2, Biobase 2.58.0, GEOquery 2.66.0, limma 3.54.0) by NCBI were used to study the differentially expressed genes (https://www.ncbi.nlm.nih.gov/geo/geo2r). The criteria used for selection were an adjusted p-value below 0.05 and an absolute log2 fold change (logFC) greater than 1.5 or smaller than -1.5. In addition, genes with logFC values over ≥ 1.5 were categorized as upregulated, whereas genes with logFC values ≤ -1.5 were categorized as downregulated. The logFC cutoff of 1.5 was chosen to ensure the identification of genes with substantial changes in expression that are more likely to have biological significance. This threshold balances sensitivity and specificity, capturing relevant DEGs while minimizing noise from minor fluctuations in gene expression. The application of Python programming facilitated the classification of genes based on their up- and down-regulation [33].

### Biomarker gene identification from DEGs

The Python program identified common genes for both up and down-regulation in the CSV file by analyzing all the GSEs that are under investigation. By selecting DEGs that are consistently observed across at least two independent datasets (GSEs), the likelihood of identifying true positive biomarkers is increased, as these genes are more likely to be relevant to dengue pathogenesis. The resulting database had symbols and names for genes that were both upregulated and downregulated [34]. The DEGs that have been identified in at least two GSEs are considered for further analysis as potential biomarkers. The ReactomeFI plugin in Cytoscape 3.10.0 (https://cytoscape.org/) was employed to examine the relationships between these genes. cytoHubba assessed the hub genes using a variety of criteria, utilizing a total of 12 distinct methodologies. The MCODE technique used in Cytoscape was utilized to detect gene clusters. The algorithm works by finding densely connected regions based on node connectivity and node weight. In this study, default parameters were used, which include a degree cutoff of 2, node score cutoff of 0.2, and k-core of 2, to ensure the detection of significant gene clusters. The study utilized at least two cytoHubba scoring techniques and considered MCODE clusters as potential biomarkers, enhancing the investigation’s overall comprehensiveness [35]. The “Top count” is at least common in two clustering methods out of 12 in cytoHubba and matches the genes with the MCODE clustering method in Cytoscape’s Software is considered a potent biomarker. The Top count designates nodes that are at the center of a network and have a high degree of connectivity; these nodes frequently serve as important regulatory factors or constituents of biological processes.

### GO and Pathway enrichment analysis of biomarkers

The pathway and GO enrichment analysis of biomarker genes was conducted utilizing a web-based Bioinformatics platform called Weishengxin bioinformatics (https://bioinformatics.com.cn/). The statistical criterion of the adjusted p-value was ≤ 0.05, along with the Benjamini & Hochberg adjustment for multiple testing. The route analysis was conducted using Enricher’s “KEGG” library, which may be accessed at (https://maayanlab.cloud/Enrichr/). The following parameters were used: adjusted p-value cutoff of ≤ 0.05, and the Benjamini & Hochberg method was applied for multiple testing correction. The GO enrichment analysis utilizes the enrichment libraries of Biological Processes, Cellular Components, and Molecular Functions [36].

### Protein-protein interaction network construction

Protein-protein interaction (PPI) network analysis is crucial for predicting the function of proteins that interact with each other. It is a viable instrument that can be utilized to comprehend cellular activity and the mechanisms of diseases [37]. The STRING database (http://string-db.org) primarily focuses on combining a comprehensive collection of known and anticipated protein-protein association data to provide a reliable evaluation of protein-protein interactions [38]. A PPI network was created using the STRING database and visualized using the Cytoscape software. After contrasting the String PPI network, three cluster methods applied MCL to find natural clusters based on the stochastic flow and DBSCAN clustering to find clusters based on the local node density, and the Klegacy clustering method was applied to identify clusters based on a combination of network topology and specific gene characteristics [39].

## Results

### Identification of DEGs

**Fig 2:**
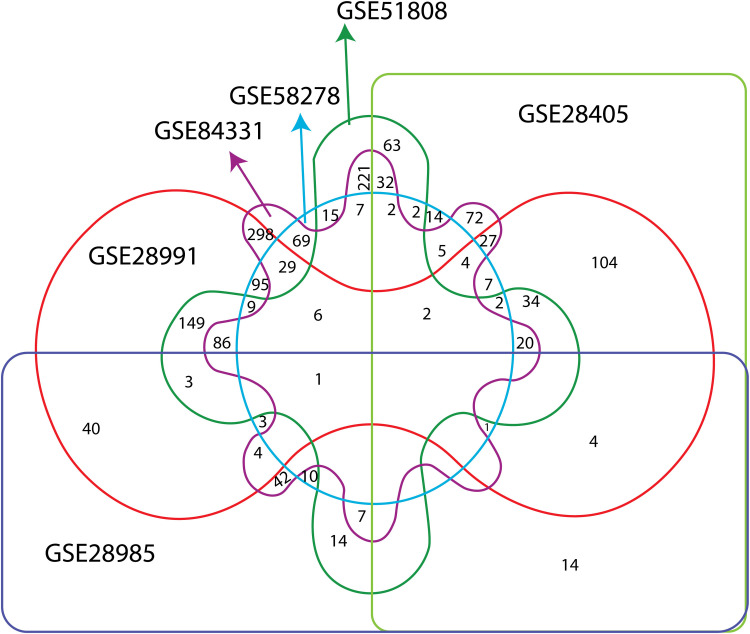
The Venn diagram illustrates the overlap of DEGs among six datasets. The GEO datasets used in the study are GSE51808, GSE58278, GSE84331, GSE28405, GSE28991, and GSE28985. Each data set is represented by a distinct colored shape, with intersections between these shapes indicating the number of common genes among the datasets. For instance, the central area where all datasets intersect includes the smallest number of common genes, reflecting the highly specific overlaps. In contrast, non-overlapping regions of each shape represent genes unique to that dataset. The numbers within each region specify the exact count of genes in that intersection. Arrows point to specific datasets, emphasizing their contributions and intersections within the broader context of gene expression analysis.

The NCBI GEO database was utilized to get six microarray datasets, namely GSE28405, GSE28985, GSE28991, GSE51808, GSE58278, and GSE84331 (Table 1). For the differential expression gene (DEG) analysis, a total of 334 samples were employed. These samples included 71 healthy individuals and 265 patients with Dengue in different cell types (Table 1). The common DEGs were identified to make the experiment more significant as the potent biomarkers should avail at least two of the experiments. A Venn diagram to visually represent the overlap of DEGs across the six datasets analyzed. This visual aid enhances the clarity of the results and allows for a more intuitive understanding of the shared and unique DEGs among the datasets. A total of 971 common genes were identified; 193 DEGs were upregulated, and 778 DEGs were downregulated (S1 Table). The Significant DEGs were further analyzed (Fig 2), and they were compared with the MCODE retrieved clusters. Among all the biomarkers, the most significant, based on the number of occurrences in cytoHubba and MCODE methods, have been retrieved and listed along with their names (Table 2).

**Table 2:** The most significant biomarker from the analyzed datasets.

Sl no	Gene Symbol	Gene Name	Experiment ID	Expression in control	Expression in cases	Log2 fold change	Regulation
1	CXCL10	C-X-C motif chemokine ligand 10	GSE28991	7.5	1.0	-6.379	Down
			GSE58278	6.8	1.2	-5.424	
2	ZWINT	ZW10 interacting kinetochore protein	GSE28991	4.0	1.9	-2.042	Down
			GSE84331	3.5	0.5	-4.217	
3	BUB1	BUB1 mitotic checkpoint serine/threonine kinase	GSE28991	5.0	1.1	-3.914	Down
			GSE84331	6.0	2.1	-3.876	
4	EXO1	Exonuclease 1	GSE28991	4.8	1.8	-2.205	Down
			GSE84331	5.0	1.6	-3.874	
5	CXCL11	C-X-C motif chemokine ligand 11	GSE28991	7.0	2.8	-3.301	Down
			GSE58278	6.5	3.1	-2.834	
6	AURKA	Aurora kinase A	GSE28991	5.5	2.5	-2.961	Down
			GSE84331	7.0	1.9	-5.304	
7	AURKB	Aurora kinase B	GSE28991	6.5	3.2	-3.269	Down
			GSE84331	6.0	3.3	-2.701	
8	CDCA5	Cell division cycle associated 5	GSE28991	5.0	2.5	-2.41	Down
			GSE84331	6.0	1.5	-5.195	
9	CDCA3	Cell division cycle associated 3	GSE28991	4.0	1.9	-2.343	Down
			GSE84331	8.0	2.0	-3.002	
10	CDC20	Cell division cycle 20	GSE28991	6.5	1.5	-4.027	Down
			GSE84331	6.5	2.8	-3.76	
			GSE28405	4.0	2.2	-2.132	
11	STAT1	Signal transducer and activator of transcription 1	GSE28991	4.0	2.2	-1.775	Down
			GSE58278	3.8	2.4	-2.339	
12	CCNF	Cyclin F	GSE28991	3.5	1.7	-1.86	Down
			GSE84331	4.0	2.2	-1.783	
13	CDK1	Cyclin dependent kinase 1	GSE28991	5.0	2.2	-1.758	Down
			GSE84331	5.0	1.0	-4.793	
			GSE28405	6.0	2.9	-2.78	
14	RFC3	Replication factor C subunit 3	GSE28991	4.5	1.9	-1.692	Down
			GSE84331	4.5	2.0	-2.506	
15	SMARCA2	SWI/SNF related, matrix associated, actin dependent regulator of chromatin, subfamily a, member 2	GSE28991	2.0	4.0	1.501	Up
			GSE84331	2.5	5.0	1.685	
16	RPL37A	Ribosomal protein L37a	GSE28991	1.5	4.5	1.742	Up
			GSE84331	2.0	5.2	1.779	
17	MYC	v-myc avian myelocytomatosis viral oncogene homolog	GSE84331	3.0	7.2	2.135	Up
			GSE58278	3.5	8.4	2.356	
18	RPL22	Ribosomal protein L22	GSE28991	2.0	5.5	1.514	Up
			GSE84331	2.5	6.3	1.995	
19	RPS23	Ribosomal protein S23	GSE28991	1.8	6.8	1.985	Up
			GSE84331	2.2	7.2	2.067	
20	LEF1	Lymphoid enhancer binding factor 1	GSE28991	2.5	9.0	2.104	Up
			GSE84331	3.0	15.0	3.629	

Differential gene expression analysis was conducted by comparing dengue patients with healthy controls across several microarray datasets obtained from the NCBI GEO database. While several genes consistently identified dysregulated in dengue patients, such as CXCL10, ZWINT, BUB1, AURKA, STAT1, etc. there was a notable variability in gene expression patterns across the datasets. Some genes exhibited consistent upregulation in all datasets, while others showed more pronounced differential expressions in specific datasets (Table 2). This variability can be attributed to factors such as differences in sample size, geographic location, and demographic characteristics of the patients. Overall, these results suggest that while certain biomarkers could serve as general indicators for dengue infection, variability across datasets needs to be considered when selecting biomarkers for clinical applications.

### GO and KEGG pathway analysis

**Fig 3:**
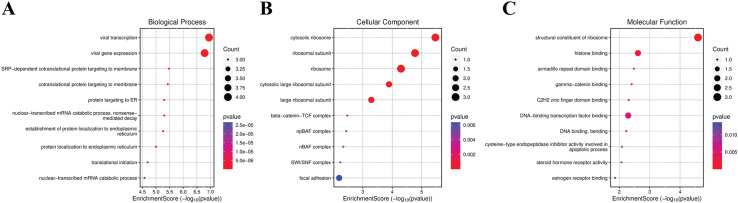
Upregulated biomarkers GO enrichment Biological Processes. (A) Dot plot of Biological Processes, (B) Cellular Component, (C) Molecular function. The dot plot serves as a visual representation of enrichment counts, employing dot size for comparison with the right-side parameter across three distinct datasets. The P value denotes the significance of the plot. The Dot size signifies the substantial involvement of most biomarker genes in these processes.

The study presents comprehensive results from GO enrichment analysis and KEGG pathway analysis of identified biomarkers. Specifically, the GO for both up-regulated and down-regulated biomarkers is detailed, highlighting their associated biological processes, cellular components, and molecular functions. Furthermore, the KEGG pathway analysis delineates the pathways influenced by these biomarkers, offering a deeper understanding of the regulatory mechanisms and biological pathways impacted during dengue fever infection. The biological processes of upregulated biomarkers are affected by the biomarker genes that are quite RNA and cell-division-oriented. Mitotic nuclear division, which encompasses nuclear chromosome segregation and chromosome segregation, is a complex process crucial for cell division. The analysis revealed a strong association with viral activities, including viral transcription and viral gene expression. Enrichment in ribosomal components such as cytosolic ribosome and ribosomal subunit suggests an increase in protein synthesis activities. Notably, structural constituents of ribosome and histone binding were among the top molecular functions, indicating a potential enhancement in ribosomal structure and chromatin interactions. Other processes include SRP-dependent co-translational protein targeting membrane and nuclear-transcribed mRNA catabolic process (Fig 3).

**Fig 4:**
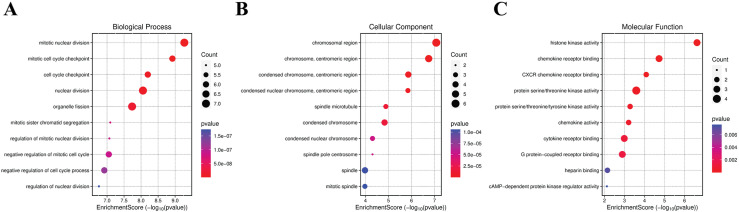
Down-regulated biomarkers GO enrichment Biological Processes. (A) Dot plot of Biological Processes, (B) Cellular Component, (C) Molecular function. The dot plot serves as a visual representation of enrichment counts, employing dot size for comparison with the right-side parameter across three distinct datasets. The P value denotes the significance of the plot. Dot size signifies the substantial involvement of most biomarker genes in these processes.

The GO analysis of downregulated genes indicated significant enrichment in processes related to mitotic nuclear division and cell cycle checkpoints, pointing towards the downregulation of cell division processes. Enriched cellular components included chromosomal regions and centromeric regions, suggesting alterations in chromosomal dynamics. Molecular functions such as histone kinase activity and chemokine receptor binding were also significant, indicating potential regulatory changes in histone modification and receptor signaling pathways. Overall, the GO enrichment results provide insights into the biological mechanisms affected by gene regulation changes, highlighting the contrasting roles of upregulated and downregulated genes in viral processes, protein synthesis, cell cycle regulation, and chromosomal dynamics (Fig 4).

**Fig 5:**
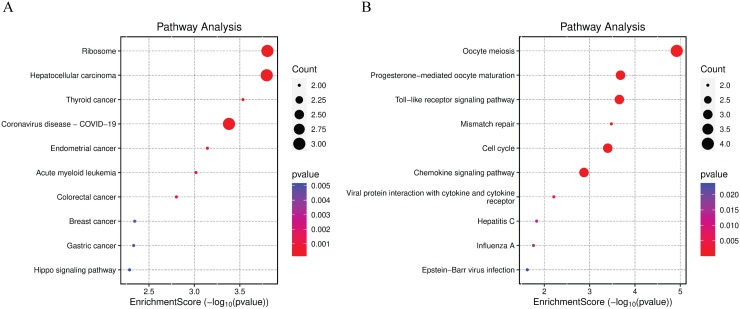
Pathway Enrichment Analysis of Upregulated and Downregulated Genes. (A) The dot plot represents enriched pathways for upregulated genes. The size of the dots indicates the count of genes, and the color gradient represents the p-value (red indicates higher significance). Notable pathways include ribosome, hepatocellular carcinoma, and thyroid cancer. (B) The dot plot represents enriched pathways for downregulated genes. The size of the dots indicates the count of genes, and the color gradient represents the p-value (red indicates higher significance). Significant pathways include oocyte meiosis, progesterone-mediated oocyte maturation, and toll-like receptor signaling pathways.

Pathway enrichment analysis for upregulated and downregulated genes reveals distinct biological pathways affected by gene regulation changes (Fig 5). The analysis shows significant enrichment in pathways related to ribosomes, indicating increased protein synthesis activity. Cancer-related pathways such as Hepatocellular carcinoma and Thyroid cancer are also highly enriched. Other notable pathways include Coronavirus disease (COVID-19), Endometrial cancer, and Acute myeloid leukemia, suggesting a potential link between these conditions and the upregulated genes (Fig 5A). Previous studies have reported similar pathways involved in immune response modulation and cell cycle regulation, which may be relevant to both Dengue and these conditions [40]. The analysis highlights significant enrichment in pathways associated with oocyte meiosis and progesterone-mediated oocyte maturation, indicating a potential impact on reproductive processes. Immune response pathways, such as Toll-like receptor signaling and chemokine signaling, are also enriched, suggesting alterations in immune regulation. Additionally, pathways related to viral infections, including Hepatitis C, Influenza A, and Epstein-Barr virus infection, show significant enrichment in pathways specifically related to Dengue infection [41,42] (Fig 5B). Overall, these results underscore the distinct biological pathways influenced by upregulated and downregulated genes, providing insights into the underlying mechanisms of gene regulation changes in various conditions.

### PPI Networking

**Fig 6:**
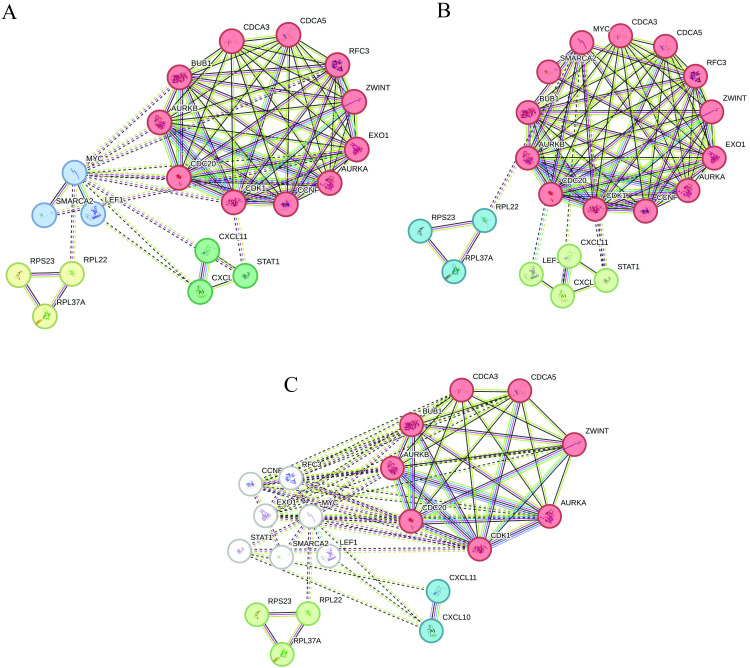
The STRING PPI Networking Clustering Method. (A) The MCL cluster method identified 5 clusters, with the most significant cluster (red) containing 17 genes such as AURKA, BUB1, and CCNB2. Other clusters include 8 genes (yellow), 5 genes (green), 3 genes (blue), and 1 gene (purple). White nodes represent non-clustered genes. (B) The DBSCAN cluster method identified 3 clusters, with the most significant cluster (red) containing 13 genes, including AURKA, BUB1, and CCNB2. The second one has 4 genes (green), and the last cluster (blue) contains 3 genes. (C) The legacy clustering method visualizes significant gene interactions corroborating the clusters identified by MCL and DBSCAN methods with 7 genes (red), 3 genes (green), 2 genes (blue), and 7 genes (white). White nodes represent non-clustered genes. The visualization highlights key gene interactions crucial for cell cycle and mitosis.

The STRING PPI network clustering methods, including MCL, DBSCAN, and Klegacy, were utilized to identify significant gene clusters based on their interactions. The MCL clustering method identified five distinct clusters. The largest cluster (red) contains 17 genes, including AURKA, BUB1, BUB1B, CCNA2, CCNB2, CDC6, CDK1, CENPE, EXO1, NEK2, NUF2, and ZWINT, which are critical for cell cycle regulation and mitotic processes. Additional smaller clusters were also identified, reflecting various biological functions (Fig 6A). Using the DBSCAN method, two main clusters were identified. The largest cluster (red) contains 13 genes, overlapping with the MCL cluster, including key genes like AURKA, BUB1, and CCNB2 (Fig 6B). This consistency across methods underscores the robustness of these gene interactions. The Klegacy clustering provided a comprehensive overview of gene interactions, highlighting similar significant clusters observed in the MCL and DBSCAN methods (Fig 6C).

## Discussion

The main contentious issue about the prognosis of dengue lies in the fact that a significant number of deaths resulting from this disease could be prevented by early detection of severe dengue and subsequent implementation of uncomplicated and cost-effective fluid replacement therapy [43]. Nevertheless, determining which individuals infected with DENV will necessitate monitoring for intravenous fluid therapy during the initial phases of illness is a challenge [44]. Frequently, individuals arrive at the hospital but are soon discharged due to their apparent good clinical condition [45]. However, within a span of two to three days, patients swiftly develop DSS, often reaching a point where treatment is no longer effective [46]. Hence, it is imperative to augment the existing clinical diagnostic methods for dengue with more sophisticated laboratory procedures that offer higher sensitivity [47]. Multiple precise diagnostic techniques can detect a confirmed infection caused by DENV, ascertain the specific serotype of the virus that the patient is infected with, quantify the amount of the virus present, and differentiate between initial and subsequent dengue infections [48]. Nevertheless, none of the existing tests can provide precise indications of the likelihood for dengue-infected patients to develop the hemorrhagic symptoms of the disease [49].

Biomarkers are crucial to disease diagnosis and treatment selection. The development of omics technologies has revolutionized biomarker identification, improving our understanding of disease mechanisms and therapeutic targets [50], broad biomarker types, systematic classification, and monitoring methods. Biomarker assessment is an analytical and clinically relevant sensing method. In silico, biomarker prediction and pathway analysis can also identify relevant genes and their associations with diseases [51]. The importance of computational methods in academic biomarker research is that they enable early detection and proactive management of health risks by understanding the complex interaction between genes and disease pathways [52].

The present study conducted a comprehensive analysis of differential gene expression in patients with Dengue fever across multiple microarray datasets retrieved from the NCBI GEO database [53]. The observed variability in gene expression between dengue patients and healthy controls emphasizes the complexity of identifying universal biomarkers for dengue. Differences in gene expression patterns were noted across the datasets, possibly due to factors such as patient demographics, viral strain diversity, or dataset-specific biases. Through the integration of bioinformatics approaches, a total of 20 potential biomarker genes were identified, with 6 genes upregulated and 14 genes downregulated. These biomarkers were further analyzed to elucidate their potential roles in Dengue fever pathogenesis and identify common regulatory pathways across datasets [54].

Among the identified biomarkers, several genes were consistently dysregulated across multiple datasets. Notably, AURKA, BUB1, BUB1B, BUB3, CCNA2, CCNB2, CDC6, CDK1, CENPE, EXO1, NEK2, ZWINT, and STAT1 were among the most significant biomarkers, appearing in multiple datasets and exhibiting consistent dysregulation patterns. These genes have previously been implicated in various cellular processes, including cell cycle regulation, mitotic checkpoint control, and chromosomal segregation, suggesting their potential involvement in Dengue fever pathogenesis [55]. Functional enrichment analysis revealed that the dysregulated genes were predominantly associated with biological processes related to the immune response to the virus, cell division, RNA processing, and immune responses. Specifically, pathways involved in mitotic nuclear division, chromosome segregation, and RNA splicing were significantly enriched, highlighting the importance of these processes in Dengue fever pathophysiology. Similar approaches have been successfully employed in previous studies for other diseases, such as in cardiovascular diseases where biomarkers like troponin and CK-MB are used for diagnosis and prognosis of myocardial infarction, and in cancer research where circulating tumor cells are identified for early detection and treatment monitoring. These precedents underscore the utility and effectiveness of biomarker-based analyses in understanding disease mechanisms and improving diagnostic and therapeutic strategies [56,57]. Another analysis of dengue biomarker identification was conducted by Xie et al. 2021 who identified 69 DEGs in dengue patients, comprising 51 upregulated and 18 downregulated genes [58]. Twelve hub genes were highlighted, with IFI44L and IFI6 emerging as potential biomarkers for dengue infection [58]. In another study of stage-related and severity-related biomarkers, they have found CD38 and ZNF595 as significant biomarkers; CD38 can distinguish different clinical stages of dengue, while ZNF595 can differentiate between dengue fever (DF) and DHF [59].

The upregulation of BUB1B, CCNA2, and CCNB2 in pathway studies is associated with the development of Human T-cell leukemia virus 1 infection and Acute myeloid leukemia. Hepatitis C infection can lead to the development of STAT1, BUB1, and CDK1, which are known to be involved in the regulation of the cell cycle, oocyte meiosis, human papillomavirus infection, and hepatitis B. Additionally, these proteins have been found to play a role in immune-related pathways, such as interferon signaling and defense responses, which highlights their importance in the host immune response to Dengue virus infection [60]. PPI network analysis revealed clusters of interconnected genes associated with Dengue fever pathogenesis [61]. These clusters included key regulatory genes involved in cell cycle progression, chromosomal stability, and immune modulation.

The study has identified several genes as potential biomarkers for Dengue, some of which align with previously reported biomarkers while others represent novel findings. For example, genes such as AURKA and CDK1 have been previously implicated in cell cycle regulation during viral infections, consistent with our results. However, the identification of novel genes like ZWINT suggests new avenues for research into the molecular mechanisms of Dengue fever [62–64]. Although the biomarkers, if validated, these genes could serve as early diagnostic markers, enabling timely intervention and reducing the risk of severe outcomes such as DHF or DSS [65].

The study provides comprehensive insights into the molecular mechanisms underlying Dengue fever pathogenesis and identifies potential biomarker genes for disease diagnosis and therapeutic targeting [66]. The differential gene expression analysis presented (Table 2) highlights the distinct expression patterns between cases and controls, substantiating the biological significance of the identified biomarkers. For instance, CXCL10 and BUB1 showed markedly reduced expression in cases compared to controls, underscoring their potential role in immune response modulation during dengue infection.

These findings align with prior studies emphasizing the critical function of chemokines and mitotic regulators in viral pathogenesis. However, the reliance on in silico methods without experimental validation means that the identified biomarkers require further experimental confirmation to establish their clinical relevance. Additionally, the use of microarray data, though widely accepted, comes with inherent limitations, such as variability in data quality and potential biases. These factors may contribute to the identification of false positives, which necessitates a cautious interpretation of the results. Dengue biomarker analysis faces significant challenges, including lack of specificity and cross-reactivity, where biomarkers such as NS1 antigen and IgM/IgG antibodies often overlap with other flaviviruses like Zika and chikungunya, leading to diagnostic inaccuracies [67]. Additionally, biomarker levels vary temporally across disease stages and are influenced by host factors such as age, immune status, and genetic background, making consistent detection difficult [68]. Resource constraints in endemic regions, combined with high costs and the absence of standardized protocols, further hinder the accessibility and reliability of biomarker-based diagnostics [11]. Future studies should aim to validate these findings using techniques such as quantitative PCR or proteomics in diverse patient cohorts.

## Conclusion

The study focused on identifying biomarker genes associated with dengue fever through the integration of differential gene expression data and the construction of functional networks. Significant biomarkers such as AURKA, BUB1, BUB3, CCNA2, CDK1, and STAT1 were identified. These biomarkers were found to be involved in critical pathways, including mitotic nuclear division, chromosome segregation, and RNA splicing, as revealed by GO enrichment and KEGG pathway analyses. These processes are crucial for understanding dengue pathophysiology. The findings underscore the importance of these biomarkers in disease progression and highlight their potential in developing targeted diagnostic and therapeutic strategies for dengue fever.

## Supporting information

S1 Table The potent common DEGs from each of the datasets after sorting. (PDF)
