# Supplementary material for: Comprehensive analysis of intervention and control studies for the computational identification of dengue biomarker genes
Source: PLoS Negl Trop Dis. 2025 Mar 18;19(3):e0012914. doi: 10.1371/journal.pntd.0012914 (PMC11918421; doi:10.1371/journal.pntd.0012914)
Supplement: S1 Table — (PDF) [file pntd.0012914.s001.pdf]

S1 Table. The potent common DEGs from each of the datasets after sorting.

Sl No.	Gene symbols	Regulation
1	SOS2, SPTLC2, CXorf21, BMX, CLEC4D, CTH, KLF5, SULT1B1, STK3, RALB, TGFBR2, ZNF568, SULF2, KBTBD7, SLC4A1, CDKN2D, RNF144B, IL6R, GPAT3, FPR2, CCR2, KIR3DL1, SCML4, PRRG4, ANXA3, GBP6, LMNB1, MRVI1, DIO2, FCGR1A, LY75, MPZL3, ISL2, PSTPIP2, PTPRS, FGFR1OP2, CARD8, KRT23, SESN3, ZBTB18, MEFV, OLFM1, ELOVL5, LGALS8, KRTAP19-6, SELENBP1, PCDH7, FSTL1, ZNF19, DSC2, TGFA, MDM1, SLC2A14, HIST1H4E, F2RL1, MLPH, PROS1, AVIL, ITSN2, DNMT3A, ZNF226, CCNJL, TCP11L2, ARSD, SSFA2, RSPH9, GAS8, PTPRE, XRN1, ADPRH, TADA2A, BICD2, PRDM5, CAMK2A, DERL3, NDUFAF4, DENND1A, TSPAN7, IGF2BP1, CCL28, MRPL3, SORD, WDHD1, UBE3D, STAP1, P4HA1, FGFBP3, GADD45GIP1, SRC, XRCC4, YTHDC1, SSB, CTSF, NUDT21, DKK3, TRIM46, NSUN7, CIT, CYP51A1, MAFF, PDE4DIP, LHX4, LGALS9C, PP7080, HSPA6, TACSTD2, CD19, GTPBP2, PFAS, ZCCHC2, PGM5, CACNA2D4, EPHX2, PASK, HSPA1B, BATF2, GSDMB, ZNF84, C12orf57, KIAA0895L, FGD4, PATJ, CSNK1E, MS4A4A, LRIF1, CCNA1, HINT3, ARHGAP25, ACACA, LILRA6, SH3PXD2A, SLC7A6, DHRS9, VAV3, ZNF702P, PTPRK, SETD6, BEX2, RIN2, FBLN7, RPL22, PPP2R3B, LMO2, SPPL2B, PIK3IP1, LGSN, C11orf31, PCBD2, GLUL, CBX7, MAGED1, BLK, MME, IL23A, FCRLA, ETV7, TRAFD1, TRIM5, FAP, NAT6, CRIP2, SERPINA1, CD68, FASN, DLEU1, TNFRSF25, IL1RN, EXOC3L1, SIGLEC7, AHNAK, ABTB2, CAMK1D, DDX60L, KRT72, SOCS1, ABLIM1, CD27, RHOBTB3, CD320, RBMS2, C2orf40, ARRB1, ITGA6, NEXN, TTC21A, CD2AP, DZIP1L, LAMA5, SSPN, HSPB9, AOC2, UAP1L1, TRIM6, DDX58, STMN3, PARM1, ERICH3, SP100, ZNF200, CACNA1I, CARD16, LDLRAP1, MEGF6, PID1, PARP12, NOL4L, VSIG1, CMKLR1, CARD17, VPREB3, USP25, BCAS4, MID2, SLC22A4, PKIA, PCGF5, FCGR2B, DLL1, LARGE1, GRAMD1B, NOL9, CCDC65, IFIT5, LILRB5, HLA-DOA, CLYBL, TSTD1, MAP4K1, SNORA70B, HNRNPA1P10, ZNF684, CRYGS, SMARCA2, KIAA0319L, MIEF2, GZMK, CD69, RPS23, MS4A1, ZNF366, CPEB2, SERPING1, CCL8, RTN1, KLRB1, DEFB1, B4GAT1, JUP, ZHX3, RUFY4, CARNS1, LILRA4, SAMD4A, TDRD7, RGL1, LILRA5, SIRPG, CCDC106, GCNT2, AP3M2, PPIA, SNRPD3, NR3C2, NELL2, DSC1, ACKR3, WDR60, TYSND1, LILRB2, P2RY14, DGKA, FBRSL1, CCRL2, MCM3AP-AS1, NCOA2, FMNL2, TRIM14, HABP4, PFKFB2, NDRG2, MEF2A, FCGBP, SIGLEC5, RFX2, F11R, HEMGN, KCND1, SLC16A10, PRKCE, BCL11B, HAUS5, TGM1, NOV, DPEP3, TMEM255A, TCN2, NSG1, RNF157, CD248, CADM4, LGALS9B, HORMAD1, EFHC2, FFAR2, USP13, AXIN2, CCDC102A, PNPT1, POMT1, LEF1, MDK, RPL37A, DCHS1, FCMR, PPP1R13B, FHIT, CR2, OBFC1, OSBPL10, HESX1, LINC01550, TOR1B, CEP68, C19orf48, ELAVL1, MOV10, SMDT1, DTX3, MAL, MAFB, CDH2, MSR1, FMNL3, CENPV, TPM2, SYNE3, CFAP58, NBL1, TFEC, CARMIL2, DANCR, SEPT4, APBA2, PKD2L1, ID3, RNFT2, FAM101B, LGALS9, TMEM8B, CACNA1A, GPR141, SLC22A16, RAB24, EPAS1, BACH2, CLDN23, IL36G, SIGLEC1, AGMAT, NTNG2, NCOA7, CMTM8, ZNF667-AS1, UBQLNL, CACNA2D3, CDC42EP2, ABCC5, ITGB5, KCTD14, ETNK1, DENND5B, EPHB2, MCM6, MND1, SNX5, UCHL1, PLK4, BUB1B, CDCA7, TIMM10, VAMP5, CBX5, FBXO5, ELL2, ELP6, MTFR2, CAV1, DSCC1, SLC7A5, FLNB, HDAC8, CHST12, MKI67, IDH2, GZMB, SKA3, ZBP1, FKBP11, SHCBP1, CCNE2, ANKHD1, DLEU2, KIF14, G0S2, INTS7, EFCAB11, CENPN, C1QB, CENPF, CCNA2, TROAP, IGLV1-44, ATAD2, CDCA2, C2, HMGB3P1, SUV39H2, DIAPH3, RAD51, MTFP1, LDLR, GBP1P1, PALM2-AKAP2///AKAP2, TTK, BAMBI, RAD51AP1, RAD54L, DNAJB11, ADA, UBE2S, PI4K2B, TUBG1, LGALS3BP, GAS6, POC1A, KNL1, KIF4A, MYL6B, CDC6, CD59, E2F7, UBE2J1, IDE, WHSC1, SMC4, CDCA8, FEN1, NCAPH, SLCO4A1, PSPH, TUBB, SCARB2, ZWILCH, CENPU, CREM, DEPDC1, EBP, UBA5, NT5DC2, E2F8, BMS1P20, FAM83D, SPC25, NRAS, ARNTL2, KLHL6, KIF18B, MTHFD1L, ANLN, SPAG5, C6orf106, PDIA6, LY6E, FRMD4A, MYC, ACOT7, DOK2, CTSC, IGSF6, SCIMP, MS4A6A, ARHGAP9, DDIAS, ERI1, TRIB1, CD1C, F13A1, KCTD12, FCER1A, CTNNAL1, C10orf128, RASGRP3, SUCNR1, GFOD1, CD1E, IGIP, PITPNC1, LEPROT, MPP6, TXNIP, TNIP1, CCDC7, JAML, ANKRD36B, IL7R, LOC107985971, LOC100289230, ZNF44, TMCC1, TAGAP, LGMN, TRAF3IP3, ZNF33A, DPEP2, SF1, NFKBIZ, ANXA11, C9orf72, LRIG2, BTG1, ZNF264, SPATA13, FYB, ZNF204P, ZNF573, POM121, LINC00282, NXPE3, MAP3K7CL, SOX6, ZFP36L1, DDX17, ADTRP, PLXDC1, NOG, SDPR, CLIP4, KANSL1L, TTC9, PIAS2, IGF1R, GPR155, FBXO32, LEF1-AS1, NAP1L3, YPEL3, ZNF395, GCC2, FNIP2, HNRNPA1, KATNBL1, N4BP2L2, FBLN5, SLC26A11, SORL1, ARHGEF40, ABCG1, C4orf32, IL1B, RBBP6, PPM1K, POLI, RBM12B, RPL36A, BNIP3, PACSIN1, MCAT, KATNAL1, ARFGEF2, AXL, COBLL1, OIP5, HELLS, DCBLD1, PDE6H, LOC81691, ST8SIA4, CADM1, FABP4, LRP8, CEP128, UPB1, CDKN1A, APOBEC3H, PHGDH, ZNF683, TRIM59, GPRIN3, XAF1, BIRC3, CD86, DTX3L, IL2RB, RRBP1, ACSL4, DFFA, CCL3, VEGFA, CDK5RAP2, FXYD6, SMC2, CCL3L3, IL15RA, ACSL3, TCF19, SAMSN1, C3orf58, BRCA1, CCDC34, HIST1H2BE, CSRNP1, DHCR24, DONSON, IFI16, MRPL4, GBP5, TYMP, ALG14, NETO2, GINS3, HNRNPLL, ATF5, IRF7, SMCO4, RACGAP1, GNB4, ACOT9, GPD2, SQRDL, PARP14, MAD2L1, UBE2T, IFNLR1, CASP7, TAP1, PSAT1, SPPL2A, CCNF, KIR2DL5A, ASF1B, POU2AF1, SAT1, SKA1, TMEM106C, LAP3, GGH, CNIH4, NCF1C, TMEM97, AURKA, CCDC167, HAVCR2, CDCA3, IFI35, MICB, NCF1B, KIR2DL4, DUT, ISG20, GADD45B, KIFC1, CFB, KYNU, MX2, MTHFD1, HIST1H2BD, NECTIN2, HELZ2, POLA1, ANXA2, CASP1, WDR34, CHAF1B, TCTEX1D2, STAT1, H1F0, SAP30, SNORD3D, SLC35F2, ZC3HAV1, MB21D1, BLVRA, IFITM1, ADM, NDC80, SP140, HIST1H4H, C3orf14, FBXO6, ABCA1, PSMA4, BLM, TMPO, VARS, RFC3, SESTD1, PTPRN2, ADRB2, LYST, RAB11FIP1, CPPED1, XCL2///XCL1, PSMB2, MICAL2, FCGR3B///FCGR3A, NHSL2, DOCK5, SNX10, CCL5, HIST1H1C, CST7, PELI1, NAMPT, GAPDH, CFLAR, ADAM8, PFKFB3, TNIP3, ARID5B, BCAT1, NQO1, C17orf96, SLAMF7, TIFA, PTGS1, MORC3, BEND7, CLEC7A, IL1RAP, ALPL, TMEM252, UBE2D3, APOL1, PML, LIN7A, TSPAN2, RNF135, CCNG2, RICTOR, CEACAM1, CD274, CASP5, MPZL1, SIPA1L2, MAK, TMOD2, FFAR3, PI3, LILRA3, RNF13, PLAUR, GUCY1B3, CDK5R1, PAICS, KPNA2, TMEM45A, SLC25A4, NUF2, HEY1, TRABD2A, OCIAD2, RSAD2, DHX58, DYRK2, CCL3L1, IFIT1, SLC25A24, TCEA3, PLSCR1, LINC00926, IFIT2, NT5E, KLHL3, KIAA1958, MT1A, ACACB, TNFSF13B, TNFAIP6, PIK3AP1, ATF3, C21orf2, ARRDC3, LAMP3, C11orf80, CXCL11, TSPAN18, SAMD9, SAMHD1, CDKN2C, GBP4, FOXC1, CCL2, C10orf35, SLA, IL27, FAR2, ZNF540, NFIL3, PLEKHB1, QKI, FAM159A, CMPK2, CXCR6, GOLGA8A, CXCR3, AIM2, TCF7, SRGAP2, GCH1, CXCL10, TMEM204, NT5C3A, C15orf48,	All

	HSD17B8, DNAAF1, TMEM140, KIAA1324, STRBP, LY9, BATF3, IL16, IL4I1, ZNF827, CCR1, OXNAD1, SLC24A1, HOOK1, FAM102A, NMT2, CTSL, LYSMD2, ALDH5A1, WDR54, HES4, SPATS2, GTSE1, CENPA, RAB30, KIAA0101, RRM2, TOP2A, PTTG1, HMMR, PBK, GINS2, MASTL, CCNE1, KIF2C, BAK1, CD38, POLE2, PRC1, DLGAP5, MCM4, ORC1, UHRF1, GMNN, WARS, PARPBP, TIPIN, FANCI, KIF23, GLDC, TPX2, DUSP5, HMGB3, CDKN3, BTG3, IFI27L1, STIL, CDC25A, VRK2, NCAPG, ZWINT, RGS1, KIF20A, MYBL2, MTHFD2, RNASE1, AURKB, CKAP2L, CDT1, UBE2C, ASPM, SLC1A4, RMI2, MZB1, EXO1, CHEK1, CEP55, KIF15, CENPM, BUB1, EMP1, MCM2, STMN1, ITM2C, NET1, CDCA5, PHF19, GALM, FABP5, NEK2, IDH1, RPS24, LAPTM4B, ZNF641, H2AFJ, C1GALT1C1, SVIL, XCL1, TNFRSF17, JCHAIN, SLC2A5, PHACTR2, FAM129A, ADAMDEC1, MX1, MT1H, MT1E, STX11, IFI44L, IDO1, MOB3C, TSC22D3, CASP4, MT2A, OAS3, OAS2, LRRN3, FAM46A, HERC5, PMAIP1, SPATS2L, MT1G, IFI6, CCR7, IFIT3, GPR68, ISG15, IFIH1, ENO2, OASL, OAS1, TNFSF10, ESPL1, MELK, TRIP13, RBBP8, FOXM1, MCM10, CDC20, CCNB2, KIF11, NUSAP1, LAG3, IFI44, BIRC5, DTL, HJURP, CDK1, EZH2, CDC45, EPSTI1, TYMS, TK1, GBP1, CTLA4, USP18, NME1, IFI27	
2	SLC25A4, POLE2, ST8SIA4, PBK, LRP8, OIP5, AXL, FABP4, ORC1, CKAP2L, HSD17B8, MCAT, ZNF683, C11orf80, SPATS2, KIF20A, KIF2C, RNASE1, GLDC, ITM2C, CADM1, MYBL2, LOC81691, LAPTM4B, MZB1, PDE6H, ARFGEF2, TPX2, IFI27L1, PHGDH, OCIAD2, UPB1, COBLL1, CDKN1A, HMGB3, HELLS, DCBLD1, CEP128, PACSIN1, KATNAL1, APOBEC3H, CD86, ZNF641, SMC2, FOXC1, DTX3L, TSPAN18, CCL3, QKI, LILRA3, SLC25A24, TRIM59, CDK5RAP2, VEGFA, GPRIN3, RRBP1, XAF1, DFFA, ACSL3, BIRC3, FXYD6, MOB3C, ACSL4, IL15RA, SVIL, KIAA1958, IL2RB, CCL3L3, CCL3L1, ACOT9, CDKN3, IFIT2, KIR2DL5A, GPD2, DHX58, UBE2C, EMP1, NEK2, IDH1, RMI2, TMPO, CD38, BAK1, RSAD2, HES4, MCM2, PARPBP, GTSE1, RNF135, DONSON, CCDC34, CHEK1, LYSMD2, CDT1, DNAAF1, TMEM140, SMCO4, SAT1, MRPL4, KIF23, IFI16, SAMD9, PHF19, WARS, C3orf14, IL27, MAD2L1, CDCA5, HIST1H2BD, CCDC167, IRF7, CFB, GALM, GBP5, NETO2, SLC35F2, STMN1, CXCL11, CCNF, SP140, NCF1B, KIF15, HIST1H4H, GADD45B, LINC00926, GINS3, NDC80, NFIL3, SKA1, LAP3, MICB, ISG20, CMPK2, STAT1, GGH, TYMP, AURKB, MB21D1, SPATS2L, UBE2T, FAM46A, IDO1, ASF1B, GCH1, GMNN, PRC1, BRCA1, IFI35, TMEM106C, HAVCR2, DUT, ABCA1, SPPL2A, TAP1, PLSCR1, CCR7, TCF19, CASP4, C1GALT1C1, GINS2, MTHFD1, MX2, IFIH1, ASPM, STIL, MTHFD2, CHAF1B, TCTEX1D2, PARP14, LIN7A, POU2AF1, BLVRA, RRM2, ZWINT, VARS, AIM2, DHCR24, FBXO6, HIST1H2BE, CXCL10, TIPIN, CASP1, CSRNP1, KIFC1, PSAT1, C15orf48, DLGAP5, CENPM, LAMP3, CDCA3, H2AFJ, CXCR6, BUB1, GNB4, FABP5, ENO2, CENPA, IFNLR1, FANCI, PSMA4, HNRNPLL, SRGAP2, AURKA, RACGAP1, SAP30, IFIT1, HELZ2, SNORD3D, IL4I1, ZC3HAV1, MASTL, WDR54, TNFAIP6, CCR1, RFC3, TMEM97, CXCR3, TMEM45A, C3orf58, PIK3AP1, BLM, DUSP5, KYNU, NECTIN2, SLC1A4, RPS24, HMMR, CNIH4, NCF1C, IFITM1, ANXA2, NT5C3A, CASP7, SESTD1, EXO1, KIR2DL4, CDC25A, SAMSN1, H1F0, LRRN3, NCAPG, ATF5, POLA1, PTTG1, WDR34, MCM4, TOP2A, UHRF1, MT1A, SQRDL, CEP55, ADM, GBP4, KIAA0101, ALG14, VRK2, CCNE1, TNFSF13B, RGS1, TIFA, TSPAN2, PSMB2, FCGR3B///FCGR3A, XCL2///XCL1, ADRB2, PTPRN2, RAB11FIP1, CPPED1, DOCK5, NHSL2, LYST, MICAL2, ADAM8, PELI1, SNX10, ARID5B, BCAT1, NAMPT, CST7, C17orf96, NQO1, GAPDH, TNIP3, PFKFB3, SLAMF7, HIST1H1C, BTG3, CCL5, CFLAR, XCL1, BIRC5, TK1, KIF11, CCNB2, NUSAP1, LAG3, CDC45, TNFRSF17, ESPL1, HJURP, FOXM1, TRIP13, MCM10, DTL, SLC2A5, TYMS, CDK1, JCHAIN, CDC20, CTLA4, NME1, MELK, RBBP8, PHACTR2, FAM129A, OAS2, ADAMDEC1, GPR68, IFI44, IFIT3, EPSTI1, OASL, GBP1, MT1E, IFI44L, MX1, OAS3, USP18, MT1H, EZH2, ISG15, TNFSF10, MT2A, MT1G, STX11, PMAIP1, IFI6, OAS1, HERC5, IFI27	Down
3	MORC3, ZNF568, TCP11L2, MOB3C, TMOD2, TMEM252, DIO2, APOL1, SCML4, PCDH7, RNF13, GBP1, CDKN2D, SULT1B1, MLPH, MAK, IL1RAP, RNF144B, KLF5, KRTAP19-6, IL6R, SESN3, OLFM1, PI3, RALB, CAMK2A, ZNF19, PLAUR, UBE2D3, ALPL, PTPRE, ZNF226, FFAR3, CEACAM1, MPZL1, DNMT3A, BICD2, CD274, ZBTB18, GUCY1B3, RICTOR, CARD8, TGFBR2, SELENBP1, LY75, FPR2, PTPRS, CASP5, CCNG2, SLC4A1, CASP4, IDO1, PML, MEFV, SIPA1L2, DKK3, CIT, HEY1, MRPL3, TSPAN7, CTSF, FGFBP3, NUF2, TRIM46, NDUFAF4, KPNA2, PAICS, CCL28, CYP51A1, LHX4, CDK5R1, MCM3AP-AS1, FHIT, APBA2, NT5E, HLA-DOA, NMT2, CCL2, NOL4L, CD248, BEX2, SAMHD1, PIK3IP1, BCAS4, PATJ, DYRK2, C2orf40, DSC1, PASK, IFIH1, AXIN2, BACH2, VSIG1, F11R, CARNS1, SLC7A6, TCEA3, SMARCA2, NR3C2, CCR7, CDKN2C, NELL2, FBRSL1, FAM102A, ZNF667-AS1, HOOK1, ABCC5, PTPRK, CTLA4, TPM2, BCL11B, RPL22, GSDMB, MEGF6, ACKR3, C11orf31, CTSL, TMEM204, SPATS2L, C10orf35, NSG1, ATF3, IL16, FCGBP, ENO2, STRBP, TNFRSF25, CEP68, LRRN3, MAL, DCHS1, C21orf2, LDLRAP1, OXNAD1, ALDH5A1, ZHX3, RPS23, DGKA, FAR2, RPL37A, FAM159A, LINC01550, HEMGN, ZNF827, SLC16A10, EPHX2, STMN3, ARRDC3, ABLIM1, ACACB, FAM101B, IL23A, BATF3, MID2, WDR60, KIAA1324, TRABD2A, SETD6, CR2, ZNF84, CCDC65, LEF1, SLA, USP18, SLC24A1, TYSND1, CBX7, LY9, FAM46A, KLHL3, ZNF540, PLEKHB1, ITGA6, TCF7, GOLGA8A, OBFC1, NDRG2, ARRB1, NET1, KLHL6, NME1, BMS1P20, RAB30, FLNB, FRMD4A, MYC, F13A1, KCTD12, CLEC7A, TSC22D3, PTGS1, BEND7	Up
